# Computation sparks chemical discovery

**DOI:** 10.1038/s41467-020-18651-x

**Published:** 2020-09-28

## Abstract

Computational chemistry methods with an optimal balance between predictive accuracy and computational cost hold major promise for accelerating the discovery of new molecules and materials. We at *Nature Communications* are eager to continue our engagement in this exciting and rapidly evolving field.

Theoretical and computational modelling is ubiquitous in materials research. Modelling can significantly help to bridge the results of fundamental materials research to actual materials production by significantly reducing timescales. The computational chemistry approaches developed over the years have been an invaluable tool to provide deep insight into chemical processes beyond what can be directly measured experimentally. A new Collection [https://www.nature.com/collections/ncomms-compchem] showcases recent progress in developing these computational frameworks.

For many years, density functional theory (DFT) was considered the method of choice to study the electronic structure of molecules, materials and condensed systems, enabling an optimal trade-off between accuracy and computational cost. This balance could be achieved by including the complex many-body electron–electron interactions within a functional of the density, i.e. the exchange and correlation functional. During the 1980s and 1990s, thei key to the huge advances achieved by molecular simulations was to develop more and more accurate quantum-mechanical approximations in order to climb the so-called Jacob’s ladder, with each rung representing increasing levels of complexity and decreasing levels of approximation to the exact exchange and correlation functional. This led to the so-called chemical modelling revolution, as highlighted by Tkatchenko in his Comment entitled *Machine learning for chemical discovery*^1^.

Considering how the world has changed with the increasing availability of curated datasets containing reliable quantum-mechanical properties of molecules and materials, and how our ability to collect big data has greatly surpassed our capability to analyze it, a completely different strategy is to think about how seemingly unrelated data and properties may impact each other, studying the hidden interconnections between them. In this vein, an alternative approach to advance the predictive capability of computational approaches is to replace the physically motivated path by a data-driven search. This has given rise to big-data-driven science, which applies machine learning (ML) techniques to molecular and materials science. While ML approaches have been in use for decades for identifying correlations from big amounts of data, only recently has the computational community started to invest tremendously in programme infrastructures based on the synergetic collaboration between materials scientists, who have experimental and theoretical expertise, and computer scientists to develop ML methods aimed at discovering new molecules and materials. Under development are ML methodologies that combine electronic structure calculations and statistical analysis tools, which when fed with increasingly available molecular big data, can serve as alternatives to standard methods to explore the vast chemical space. In these ongoing efforts, the computational community currently faces theoretical and technical challenges.

Computational studies of chemical processes taking place over extended size and time scales must balance computational cost and accuracy: electronic structure methods are very accurate but computationally expensive, while atomistic models such as force fields—although computationally affordable—lack transferability to new systems.

In *Approaching coupled cluster accuracy with a general-purpose neural network potential through transfer learning*, Smith et al.^2^ discuss that an ideal solution to achieve the best of both approaches lies in developing a general purpose neural network potential that approaches CCSD(T) accuracy (coupled cluster considering single, double, and perturbative triple excitations), the gold standard in quantum chemistry, yet exhibits transferability over a broad chemical space. Most importantly for practical calculations, the resulting potential is an attractive alternative to DFT approaches and standard force fields: it is broadly applicable for conformational searches, molecular dynamics, and the calculation of reaction energies and is billions of times faster than CCSD(T) calculations.

Within traditional DFT modelling, seeking to increase the non-locality of the exchange and correlation functional in the effort to achieve more accurate approximations comes at a steep increase in computational cost, making related computational efforts impractical. A different approach in this area is to develop specialized ML functionals, whose overall accuracy does not significantly degrade when used outside their training scope.

Dick and Fernandez-Serra in *Machine learning accurate exchange and correlation functionals of the electronic density* tackle this problem by introducing a fully machine-learned functional that depends explicitly on the electronic density and implicitly on the atomic positions^3^. It approaches the accuracy of high level quantum chemistry methods at an affordable computational cost. Although these functionals were created for a specific dataset and hence are not universal, they exhibit promising transferability from the gas to condensed phase and from small to larger molecules within the same type of chemical bonding.

One common feature of machine learning approaches used in molecular simulations is that since the electronic properties are learned from quantum chemistry data, each individual model is typically limited to exploring these specific properties. Since all the physical and chemical features of a hypothetical compound can be derived by its ground-state electronic wavefunction, one way to circumvent this problem is to establish a direct link between ML and quantum chemistry with a ML model that predicts the ground-state wavefunction, as discussed by Schütt et al. in *Unifying machine learning and quantum chemistry with a deep neural network for molecular wavefunctions*^4^. The deep learning approach introduced by these authors provides full access to the electronic properties needed for practical calculations of reactive chemistry, such as charge populations, bond orders, and dipole and quadrupole moments, at a force-field-like efficiency. Moreover, the approach may enable property-driven chemical structure exploration, suggesting promise towards inverse-chemical design.

Although acknowledging the rapid evolution of computational techniques is exciting, this is not to suggest that traditional deep quantum chemistry expertise is obsolete: on the contrary, standard high-level theoretical approaches are still indispensable for solving fundamental problems in computational chemistry. A nice example is shown by Liu et al.^5^ in The electronic structure of benzene from a tiling of the correlated 126-dimensional wavefunction. Using high-level correlated wavefunction theory, the authors revisit the electronic structure of benzene, which has been a test bed for competing theories throughout the years. In alternative to the traditional description of the electronic structure in terms of molecular orbital (MO) theory, the authors rely on a method to identify and visualise wavefunction tiles, known as dynamic Voronoi Metropolis sampling. The use of such high-level theory enables them to reveal the fundamental effect of electron correlation in benzene and show its manifestation in the preference for staggered Kekulé structures, whereas the interpretation of electronic structure in terms of MO theory ignores that the wavefunction is anti-symmetric upon interchange of like-spins.

ML algorithms and natural language processing approaches also offer new possibilities in optimizing and automating reaction procedures. On-demand synthesis of small drugs is of key interest in this area, where both the forward synthesis (given a set of reactants, predict the products) and the retrosynthesis (given a target, predict reactant and reagents) can strongly benefit from recent modelling advances. Reaction predictions are usually considered a machine translation problem between simplified molecular-input line-entry system (SMILES) strings (a text-based representation) of reactants, reagents, and the products. The ultimate goal is to implement human-refined chemical recipe files to feed a robotic platform, which then execute the actual synthesis in an automated manner. A challenge here revolves around the need to extract chemical instructions from patents and the scientific literature, where they are reported in prose, and convert them to a machine-readable format. In *Automated extraction of chemical synthesis actions from experimental procedures*, Vaucher et al.^6^ make a first important step towards implementing the automated execution of arbitrary reactions with robotic systems by developing a deep-learning model that performs the conversion of chemical instructions for organic synthesis reactions.

Although data-driven computational approaches clearly hold promise towards speeding up the discovery of new molecules and materials, at the moment current applications are only at the beginning of the exploration phase. The reliability of any ML approach depends on the availability of extensive datasets for model training, the bottleneck in cases where data is not abundant or difficult to generate. Along with the need for extensive curated data sets of microscopic and macroscopic molecular properties, future work should target the development of more transferable models with universal approximations that can treat local chemical bonding and non-local interactions on the same foot.

As an ultimate goal, the hope is to develop ML approaches that can not only provide predictive models but also interpretable models to stimulate the formation of novel scientific concepts and deeper understanding of a given research field, as Häse et al. suggest in their Perspective piece *Designing and understanding light-harvesting devices with machine learning*^7^.

We at *Nature Communications* are eager to continue our contribution to this exciting and fast-developing field. While we acknowledge the importance of standard high-level computational frameworks, we recognize the tremendous potential of data-driven ML schemes towards accelerating the discovery of material systems with target properties. We strongly believe that a synergistic effort across disciplines—involving computational chemists, computer scientists, experimental chemists and material scientists—will play a crucial role for enhancing the rational design of new molecules and materials.

“While we acknowledge the importance of standard high-level computational frameworks, we recognize the tremendous potential of data-driven ML schemes towards accelerating the discovery of material systems with target properties.”
